# Evaluation of the Therapeutic Effect of a Bovine-Derived *Klebsiella pneumoniae* Phage Endolysin on Mouse Mastitis Models

**DOI:** 10.3390/microorganisms14081686

**Published:** 2026-07-31

**Authors:** Zhuangkan Yan, Zhaomin Wu, Jieru Zhang, Yan Zeng, Chenfei Ma, Yin Wang, Zexiao Yang, Yixin Huang, Zhengzhong Luo, Kang Yong, Suizhong Cao, Xueping Yao

**Affiliations:** 1 College of Veterinary Medicine, Sichuan Agricultural University, Chengdu 611130, China; 2 Sichuan Key Laboratory of Agricultural Animal Diseases and Veterinary Public Health, Chengdu 611130, China; 3 Lanzhou Institute of Husbandry and Pharmaceutical Sciences, Chinese Academy of Agricultural Sciences, Lanzhou 730050, China; 4 Chongqing Three Gorges Vocational College, Chongqing 402160, China

**Keywords:** *Klebsiella pneumoniae*, phage therapy, endolysin, mastitis

## Abstract

Multidrug-resistant hypervirulent *Klebsiella pneumoniae* caused by the improper use of antibiotics has become a major threat to the dairy industry and public health, posing a major challenge to the treatment of bovine mastitis. The resurgence of phage (and its lysozyme therapy) provides a novel strategy to combat such infections. In this study, bovine *Klebsiella pneumoniae* phage vB_Kpn_B01 nucleic acid was used as a template to successfully achieve the prokaryotic expression of its endolysin. The results of protein gene annotation and prediction showed that Lysin K had the function of lyase, and was designated Lysin K. After optimizing the production conditions, Lysin K could be produced in a soluble form after induction with 0.25 mmol/L IPTG at 37 °C for 16 h, and the concentration was 100 μg/mL after purification. The stability test confirmed that Lysin K can maintain stable antibacterial activity over a wide temperature and pH range. The minimum inhibitory concentration (MIC) of Lysin K was 0.01 μg/mL and remained stable in milk. The Lysin K showed strong lysis activity in vitro and could effectively lyse three types of multidrug-resistant hypervirulent *Klebsiella pneumoniae*. In the mouse mastitis model induced by *Klebsiella pneumoniae* KP18 (10^8^ CFU/mL), the intervention of Lysin K can significantly reduce the bacterial load, alleviate the inflammatory response, and restore the normal functional structure of the target mammary gland. In summary, Lysin K has valuable application prospect in the prevention and treatment of bovine mastitis caused by drug-resistant *Klebsiella pneumoniae* and the development of phage lysin preparations.

## 1. Introduction

*Klebsiella pneumoniae*, an opportunistic pathogen of the family Enterobacteriaceae Trevisan [1], is a major causative agent of moderate to severe bovine mastitis [2]. It can lead to reduced milk production, deteriorated dairy quality, and even animal death [3]. In the United States alone, mastitis causes annual economic losses exceeding 2 billion US dollars [4,5]. Furthermore, this bacterium is a common cause of drug-resistant opportunistic infections in hospitalized patients, triggering urinary tract infections, pneumonia, liver abscesses, and other disorders [6]. At present, antibiotics remain the primary treatment for diseases such as mastitis induced by *Klebsiella pneumoniae* [7]. Nevertheless, multidrug-resistant (MDR) and hypervirulent *Klebsiella pneumoniae* strains are spreading at an increasingly rapid rate across regions [8]. Widespread resistance to β-lactams and carbapenems is emerging among *Klebsiella pneumoniae* isolates [9]. In addition, improper antibiotic administration drives the continuous accumulation of plasmid-encoded antibiotic resistance genes (ARGs) in *Klebsiella pneumoniae*, facilitating the emergence of extensively drug-resistant (XDR) strains harboring broad-spectrum resistance determinants [10]. All the aforementioned issues pose severe challenges to the prevention, control, and clinical treatment of *Klebsiella pneumoniae* infections

Bacteriophages are viruses that specifically infect host bacteria [11]. According to the mechanism by which they infect bacteria, they are categorized as lytic or lysogenic bacteriophages. Lytic phages can substitute antibiotics in the clinical treatment of bacterial diseases because they directly infect host bacteria, replicate, and cause bacterial lysis [12,13]. Bacteriophage endolysins are peptidoglycan hydrolases encoded by phages [14,15] that exhibit strong bactericidal activity, high safety, and low propensity for inducing bacterial resistance, thus holding great promise for therapeutic applications. However, the outer membrane of Gram-negative bacteria can hinder endolysin from approaching the peptidoglycan layer. Recent studies indicate that outer membrane permeabilizers, such as EDTA, can improve endolysin diffusion [16]. Other strategies, such as engineering fusion proteins by linking endolysins with outer-membrane-penetrating molecules like antimicrobial peptides [17], can overcome this barrier and improve bactericidal efficacy.

In this study, a novel endolysin was identified from bovine *Klebsiella pneumoniae* bacteriophages isolated from drinking water on dairy farms. The bioinformatic structural prediction and prokaryotic expression of this endolysin were subsequently performed. In vitro assays were carried out to characterize its thermal stability, pH stability, and minimum inhibitory concentration (MIC). The synergistic antibacterial effect of EDTA combined with the endolysin was also investigated, followed by simulated application tests in milk. Furthermore, the in vivo therapeutic efficacy of the endolysin was evaluated by detecting the bacterial load, IL-6 concentration, and pathological alterations in *Klebsiella pneumoniae* KP18 (10^8^ CFU/mL)-challenged mouse mastitis models after treatment. In summary, this study systematically explored the in vitro and in vivo antibacterial activities of the endolysin. As a novel antibacterial agent, this protein could substantially reduce contamination caused by *Klebsiella pneumoniae* in farm environments, thereby safeguarding the safety of livestock farms and dairy products. Meanwhile, the endolysin exhibits promising potential as an innovative antibacterial candidate for the prevention and treatment of bovine mastitis induced by drug-resistant *Klebsiella pneumoniae*, and provides theoretical support and an alternative strategy to facilitate the implementation of antibiotic reduction policies.

## 2. Materials and Methods

### 2.1. Strains and Plasmids

All strains and bacteriophages used in this study were preserved in the Animal Quarantine Laboratory of Sichuan Agricultural University. *E. coli* DH5α, *E. coli* BL21 (DE3), pET-32a (+) vector, and cloning vector T-Vector pMD19 (Simple) were purchased from Takara Biotechnology (Dalian) Co., Ltd. (Dalian, China).

### 2.2. Main Reagents

TaKaRa MiniBEST Viral RNA/DNA Extraction Kit Ver.5.0 was purchased from Takara Bio Inc. (Dalian, Liaoning, China). Mouse IL-6 ELISA Research Kit was obtained from Jiangsu EIA Industrial Co., Ltd. (Yancheng, Jiangsu, China). LB medium was purchased from Qingdao Haibo Bio., Ltd. (Qingdao, Shandong, China). Agar powder, PBS powder (phosphate buffer solution), SDS-PAGE gel kit, and pre-stained protein molecular weight marker were purchased from Beijing Solarbio Science & Technology Co., Ltd. (Beijing, China). Oriscience Plasmid Mini Extraction Kit and microsyringe (100 μL) were obtained from Chengdu Haobo Technology Co., Ltd. (Chengdu, Sichuan, China). Hypur T Ni-NTA 6FF (His-tag) gravity column were purchased from Sangon Biotech (Shanghai) Co., Ltd. (Shanghai, China).

SM buffer [18] was prepared as follows: 5.8 g of NaCl, 2 g of MgSO_4_·7H_2_O, 50 mL of 1 mol/L Tris-HCl (pH 7.5), and 5 mL of 2% gelatin (m/V) were dissolved in distilled water to a final volume of 1000 mL. The solution was autoclaved at 121 °C for 20 min and stored at 4 °C.

### 2.3. Sequence Analysis

A genome annotation of the sequenced bacteriophage vB_Kpn_B01 (GenBank accession number MT380195.1) was performed, and ORF36 was predicted to possess lytic enzyme activity, which was designated Lysin K. SnapGene (version 6.0.2) software was used to translate the nucleotide sequence of this lysin and analyze its physicochemical properties, including the molecular weight, amino acid composition and signal peptide of the encoded protein. Protein sequences homologous to the amino acid sequence of the lysin in this study were downloaded from the alignment results generated by the NCBI database (https://www.ncbi.nlm.nih.gov/). The online analytical tool CD-Search from NCBI (https://www.ncbi.nlm.nih.gov/Structure/cdd/wrpsb.cgi, accessed on 28 April 2025) was applied to predict the conserved domains of the lysin.

### 2.4. Expression Vector Construction

Using the phage *vB_Kpn_B01* genomic DNA as a template, the target fragments of Lysin K and Holin flanked by *EcoR I* and *Hind III* restriction sites were amplified, then 10 μL of 2× Taq Master mix was added, followed by a 10 min extension at 72 °C to add ploy (A). The gel extraction product was ligated with T-Vector pMD19 (Simple) overnight. The ligation product was transformed into *E. coli* DH5α competent cells and verified by M13 primers. The PCR products were ligated into pMD19T and sequenced by Chengdu Qingke Biotechnology Co., Ltd. (Chengdu, Sichuan, China). Positive clones pMD19T-Lysin K and pMD19T-Holin were confirmed by sequencing. The pMD19T-Lysin K, pMD19T-Holin, and pET32a (+) were digested with EcoR I and Hind III, and the products were ligated by T4 DNA ligase overnight at 16 °C. The ligation products were transformed into E. coli BL21 (DE3) competent cells, and pET32a-Holin was used as a plasmid construction control. The positive transformants pET-32a-Lysin K and pET-32a-Holin were cultured and preserved respectively, and pET-32a-Lysin K was used for subsequent protein production. Primers used for construction are listed in Table 1.

### 2.5. Induced Expression, Purification and Concentration

The pET-32a-Lysin K positive transformant and pET-32a (+) empty vector were inoculated at 1% volume fraction into LB containing 50 μg/mL ampicillin to the logarithmic growth phase, and 1 mL of uninduced culture was retained. The remaining part was added with a final concentration of 1.0 mmol/L IPTG and induced overnight at 16 °C. The induced culture was centrifuged at 8000 r/min for 5 min, and the bacterial precipitate was resuspended and washed with 100 μL sterile PBS (repeated 3 times). Subsequently, the bacterial precipitate was resuspended in 80 μL PBS and mixed with 20 μL 5 × SDS-PAGE loading buffer for SDS-PAGE analysis. Uninduced cells and empty vectors were used as controls to initially evaluate protein production levels. The positive expression strains were collected by centrifugation (8000 r/min, 5 min) after amplification and induction. After three instances of PBS washing, the cells were ultrasonically disrupted in an ice water bath until clarified. After centrifugation at 4 °C and 8000 r/min for 10 min, the supernatant was collected by 0.22 μm filter. The pellet was resuspended in PBS to the same volume as the supernatant for comparative SDS-PAGE analysis, and the protein solubility was analyzed by SDS-PAGE. According to the results of soluble analysis, the expression conditions were optimized by the above method (Table 2), and the expression was induced under the optimal expression conditions. The bacterial supernatant was purified by Hypur T Ni-NTA 6FF (His-tag) (purchased from Sangon Biotech (Shanghai) Co., Ltd., Shanghai, China) gravity column, and then 15 mL Millipore ultrafiltration tube was used at 4 °C, 5000 r/min (accelerated 5 times, decelerated 5 times), under the condition of filtration for 30 min per cycle to concentrate, collect protein samples, perform SDS-PAGE analysis, and use 6× His antibody for western blotting detection.

### 2.6. In Vitro Antibacterial Activity Assay

#### 2.6.1. Cleavage Spectrum of Lysin K

The cleavage spectrum of Lysin K was determined using the spot-lawn method. Sterilized 0.6% semi-solid LB agar was melted by heating and cooled to 60 °C. A total of 3 mL of the agar medium was mixed with 200 μL of the tested bacterial suspension, and the mixture was poured into sterile disposable Petri dishes. After the medium solidified, 10 μL of Lysin K solution (100 μg/mL) was spotted onto the lawn, followed by overnight incubation at 37 °C. Information of all tested bacterial strains is listed in Table 3.

#### 2.6.2. Enzymatic Characteristics of Lysin K

The lytic activity of protein production products was determined via the turbidity method as described by Wang [19]. A 200 μL reaction system was adopted, consisting of 100 μL bacterial suspension mixed with 100 μL sample for activity testing. The bacterial suspension was prepared as follows: bacterial cultures grown to the logarithmic growth phase were centrifuged at 8000 r/min for 2 min, and the pellet was resuspended in PBS buffer and diluted to an OD_600_ of 2.0 ± 0.1 (this resuspension solution was used in all experiments unless otherwise specified). Detailed information for each experimental group is listed below.

Thermal stability group: 100 μL Lysin K solution (100 μg/mL) was incubated with 100 μL KP18 bacterial suspension at different temperatures ranging from 10 °C to 45 °C.

pH stability group: 100 μL Lysin K solution (100 μg/mL) was mixed with 100 μL KP18 bacterial suspension, and the pH of the reaction system was adjusted to 3, 5, 7, 9 or 11.

MIC determination group: 100 μL Lysin K serially diluted at concentrations from 100 μg/mL to 0.0001 μg/mL was mixed with 100 μL KP18, LKP and BKP bacterial suspension.

All reaction systems were incubated in sterile 96-well plates for 2 h, and all experiments were performed in three biological replicates. A turbidity blank control group was set up within each group, in which 100 μL sterile PBS was mixed with 100 μL bacterial suspension and incubated under identical conditions. After 2 h of incubation at 25 °C (this temperature was used for all assays unless otherwise specified), the OD_600_ value of each well was measured. The turbidity reduction rate was calculated according to the following formula:

Turbidity reduction rate = (OD_600_ of blank control group − OD_600_ of experimental group)/OD_600_ of blank control group × 100%

#### 2.6.3. Effect of EDTA on the Antibacterial Activity of Lysin K

The Oxford cup method and the turbidity method described in Section 2.6.2. were used to detect the antibacterial activity of endolysin Lysin K alone and EDTA+Lysin K against *Klebsiella pneumoniae*. A total of 30 mL sterilized LB medium was heated to complete melting, cooled to 55 °C, mixed with 500 μL *Klebsiella pneumoniae*, and poured into petri dishes until complete solidification. In a sterile ultra-clean bench, Oxford cups were placed vertically on the medium surface with sterile disposable tweezers and gently pressed to ensure tight contact without gaps. After the agar was completely dried and solidified, the Oxford cups were removed. The grouping and sample addition in the medium are shown in Table 4.

#### 2.6.4. Determination of Time-Kill Curves of Lysin K

The 100 μL Lysin K solutions at concentrations of 200 μg/mL, 100 μg/mL and 50 μg/mL were individually mixed with 100 μL KP18 bacterial suspension. All reaction systems were incubated in sterile 96-well plates for 4 h, and the OD_600_ absorbance was measured at time points of 0 h, 1 h, 2 h, 3 h and 4 h. A turbidity blank control was included in each treatment group, consisting of 100 μL sterile PBS mixed with 100 μL bacterial suspension. All experiments were conducted in three biological replicates. The turbidity reduction rate was calculated by the formula shown below:

Turbidity rate = (OD_600_ value of experimental group/OD_600_ value of blank control group) × 100%

#### 2.6.5. Lytic Activity in Milk

Pasteurized whole milk (Inner Mongolia Yili Industrial Group Co., Ltd., Hohhot, Inner Mongolia, China) was inoculated with *Klebsiella pneumoniae* KP18 at a concentration of 3 × 10^8^ CFU/mL at 0 h. Simultaneously, purified Lysin K at concentrations of 50 μg/mL, 12.5 μg/mL and 2 μg/mL, as well as Lysin K at the corresponding concentrations supplemented with 0.5 mmol/L EDTA, was immediately added to the inoculated milk separately. The mixtures were incubated at 37 °C for 4 h, and samples were collected every 1 h for colony counting. All experiments were performed in triplicate.

### 2.7. Establishment of a Mouse Model of Mastitis

#### 2.7.1. Animal Grouping and Infection

Twenty-four Kunming mice with 7–10 days of lactation after delivery were randomly divided into 4 groups (A, B, C, D), as shown in Table 5. All mice were anesthetized by an intraperitoneal injection of 10% chloral hydrate at 0.05 mL/kg. The 4th pair of nipples and surrounding skin were disinfected; the nipple base was fixed under a microscope with tweezers, and 2 mm of the nipple tip was excised to expose the mammary duct orifice. A 100 μL microsyringe with a blunt-tipped 30-G needle was inserted into the duct for approximately 1 cm, and 100 μL bacterial suspension or sterile PBS was slowly injected. Mice in each group were housed individually to avoid cross-contamination. Body weight, mental state, feeding status, and appearance changes of mammary glands and surrounding tissues were observed and recorded.

#### 2.7.2. Bacterial Load and IL-6 Concentration in Mouse Mammary Glands

Subsequently, 24 h after injection, mouse mammary tissues were collected in a sterile ultra-clean bench, and pathological changes were observed and recorded. The 4th pair of mammary glands was harvested: one side was immersed in 10% formaldehyde solution for subsequent histopathological section preparation, while the other side was used for bacterial load detection based on the plate count method. The concentration of cytokine IL-6 in mammary glands was detected using a mouse IL-6 ELISA Research kit was obtained from Jiangsu EIA Industrial Co., Ltd. (Yancheng, Jiangsu, China).

#### 2.7.3. Preparation of Histopathological Sections

Mammary gland tissues fixed with 10% formaldehyde were removed, trimmed, and processed through rinsing, dehydration, clearing, wax immersion, embedding, sectioning, and staining. Epithelial cell morphology and arrangement in mammary gland histological sections were observed under a microscope.

### 2.8. Therapeutic Evaluation of Lysin K Against Mastitis in Mice

Based on the modeling results in Section 2.7, mouse mammary glands were challenged with 10^8^ CFU/mL *Klebsiella pneumoniae* suspension for model establishment. Thirty mice with successful mastitis modeling were randomly divided into 5 groups (6 mice per group), as detailed in Table 6. Group A (Lysin K+EDTA group) was set to verify the toxicity of Lysin K and EDTA to mouse mammary tissues. Prepared samples were injected intramammarily at a volume of 100 μL per nipple into the mammary glands of mice in Groups A–E that had been challenged with *Klebsiella pneumoniae* KP18 for 24 h, following the procedure described in Section 2.7.1. After injection, mice were placed in a supine position in a ventilated area to recover naturally and housed individually to avoid cross-contamination. Mice were humanely euthanized via cervical dislocation under isoflurane anesthesia 24 h after injection. Mammary gland histological sections were prepared as described in Section 2.7.3, and the therapeutic effect of endolysin Lysin K on the mouse mastitis model was evaluated using IL-6 concentration, bacterial load, and the severity of mammary gland lesions as indicators.

### 2.9. Statistical Analysis

Statistical analysis was performed using GraphPad Prism 7.0. Significant differences were analyzed by two-way analysis of variance (ANOVA), and *p* < 0.05 was considered statistically significant among groups.

## 3. Results

### 3.1. Sequence Analysis of Endolysin Lysin K

The gene sequence of endolysin Lysin K was imported into SnapGene software for translation into the amino acid sequence, followed by physicochemical property analysis. The full-length LysK gene is 414 bp, encoding a protein of 137 amino acids with a theoretical molecular weight of approximately 15.3 kDa. Signal peptide prediction via the software indicated that Lysin K contains no signal peptide sequence. Conserved domain prediction using CD-search revealed the presence of three catalytic domains within Lysin K (Table 7): an L-Ala-D-Glu_peptidase_like domain spanning amino acids 7–390, a D-alanyl-D-alanine carboxypeptidase domain located at residues 55–225, and a Peptidase_M15 domain covering amino acids 7–363. All three domains correspond to catalytic regions of lytic enzymes, while no substrate-binding domains were identified in the sequence.

### 3.2. Plasmid Construction

**Figure 1:**
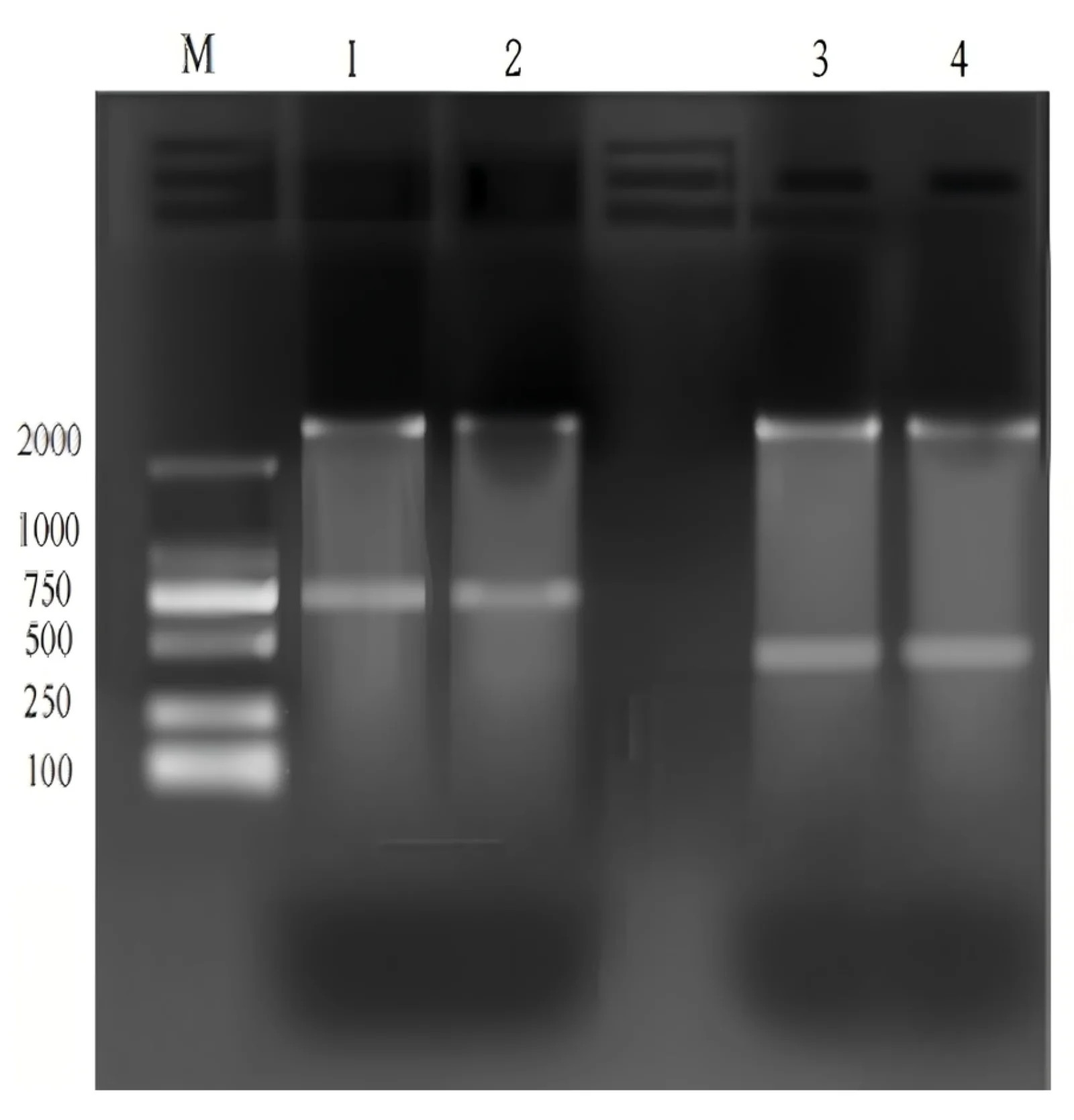
Double enzyme digestion of recombinant plasmid pMD-19T-lysin and pMD-19T-holin. M, DL 2000 DNA Marker; lanes 1–2, double enzyme digestion of pMD-19T-Holin control; lanes 3–4, double enzyme digestion of pMD-19T-Lysin K.

The recombinant T-vector plasmids, pMD-19T-Lysin K and pMD-19T-holin, were verified by double digestion. The obtained digestion fragments corresponded to the expected sizes of the lysin (414 bp) and holin (663 bp) genes, respectively. And DNA sequencing confirmed the correctness of the inserted sequences (Figure 1).

**Figure 2:**
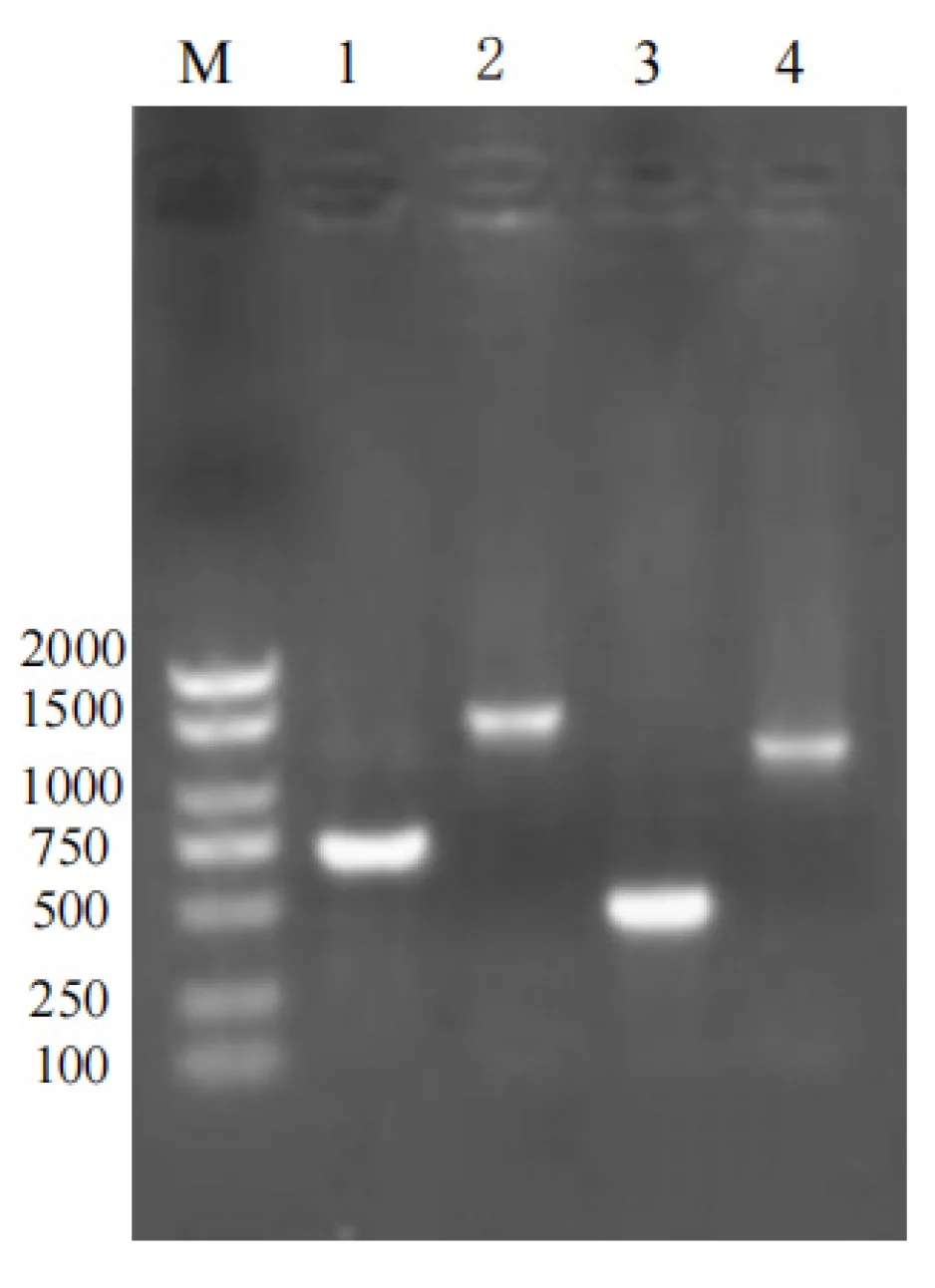
PCR verification of the recombinant expression plasmid. M: DNA Marker (D 2000 II); lanes 1: holin control; lanes 2: T7-tagged holin control; lanes 3: Lysin K; lanes 4: T7-tagged Lysin K.

The recombinant expression plasmids pET-32a-Lysin K and pET-32a-holin were first verified by PCR with target-specific primers, which produced bands of 414 bp and 663 bp, respectively. Additionally, PCR amplification using T7 primers yielded products of approximately 1500 bp and 1300 bp, confirming the correct insertion of the lysin and holin genes into the vector (Figure 2).

**Figure 3:**
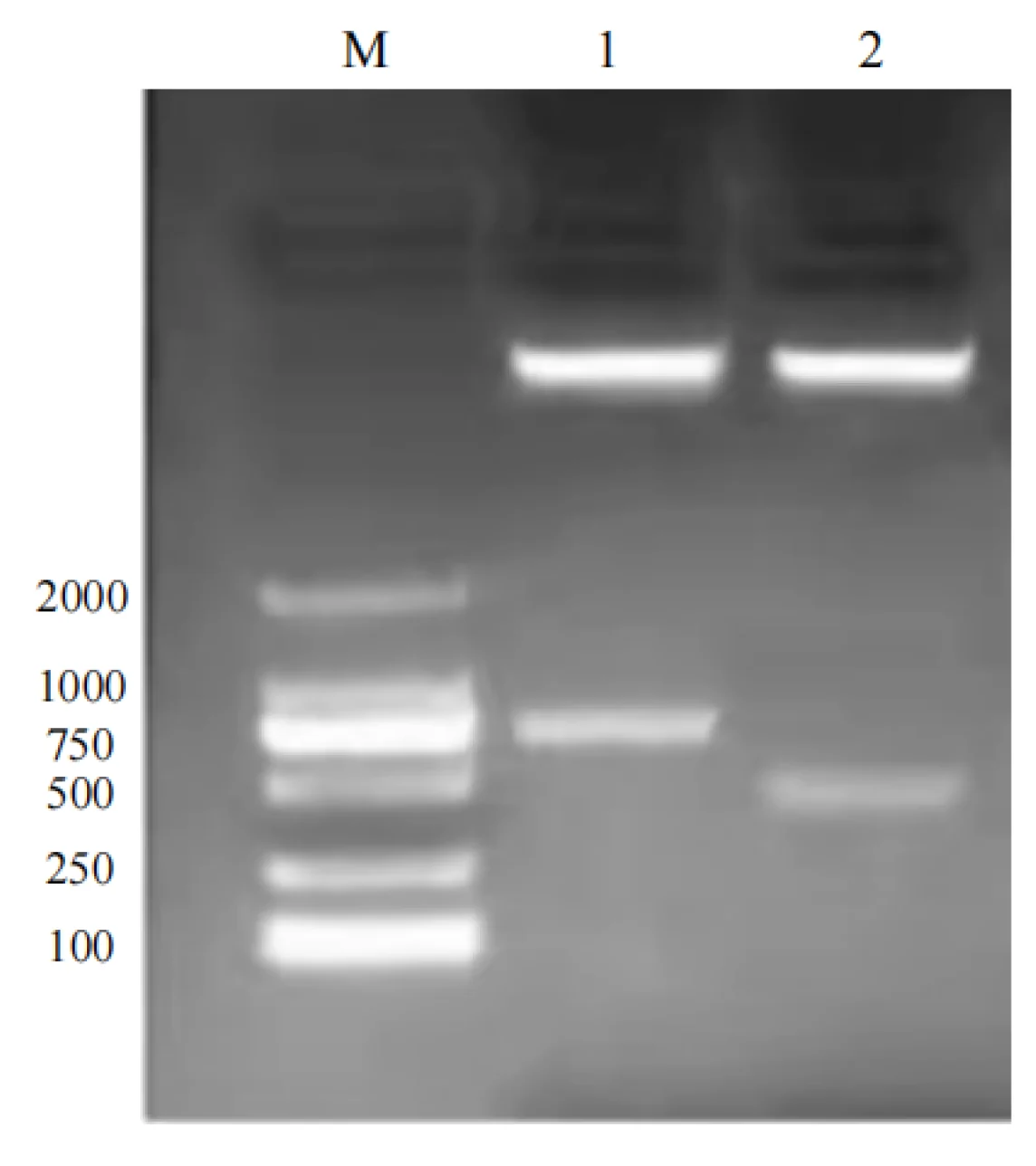
Identification of recombinant expression plasmids by double digestion. M, DL 2000 DNA Marker. lanes 1. Double enzyme digestion plasmid pET-32a—holin control; lanes 2. Double enzyme digestion plasmid pET-32a—Lysin K.

Double digestion with EcoRI and HindIII yielded fragments of 414 bp and 663 bp (Figure 3), corresponding to the Lysin K and holin genes, respectively.

### 3.3. Optimization of Expression Conditions, Solubility Analysis and Purification

**Figure 4:**
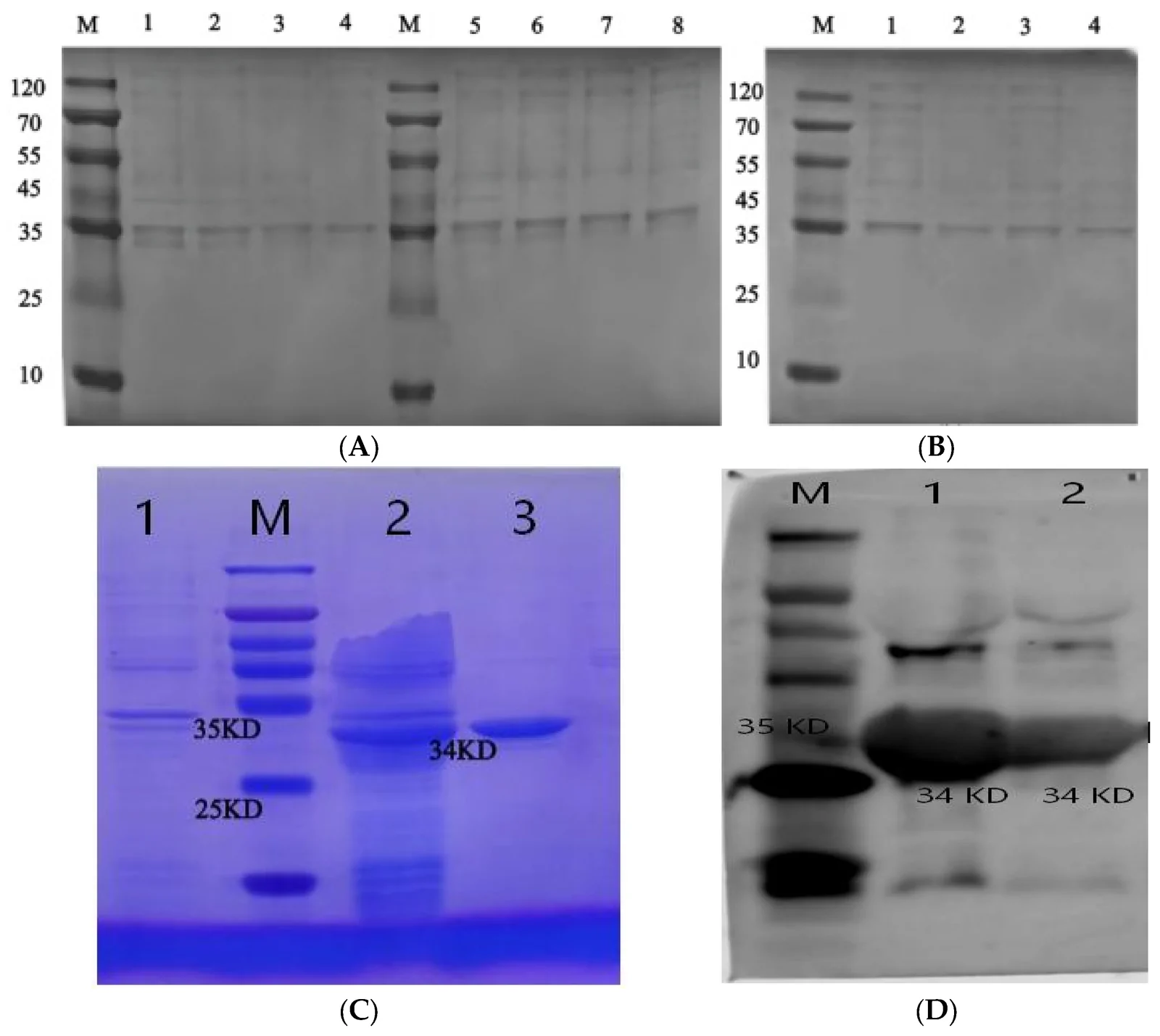
Optimization of expression conditions and purification. M. protein molecular weight marker. (**A**) Lanes 1–4: pET32a-Lysin/BL21 cultures induced with 1 mmol/L IPTG for 6, 4, 2, and 1 h, respectively; lanes 5–8: pET32a-Lysin/BL21 cultures induced with 1 mmol/L IPTG for 4 h at 37 °C, 30 °C, 25 °C, and 16 °C, respectively. (**B**) Lanes 1–4: pET32a-Lysin/BL21 cultures induced with 0.6, 0.8, 1.0, and 1.2 mmol/L IPTG, respectively, for 4 h at 37 °C. (**C**) Expression of the endolysin Lysin protein. lanes 1, pET32a(+) empty vector culture; lanes 2, supernatant of the pET32a-Lysin/BL21 bacterial lysate; lanes 3, purified supernatant of the pET32a-Lysin/BL21 bacterial lysate. (**D**) Western blot analysis. Lanes 1-2: Purified Lysin K + Recombinant Anti-beta Actin antibody (Mouse mAb).

Optimal expression conditions for Lysin K were determined to be induction with 1.2 mM IPTG at 37 °C for 6 h (Figure 4A,B), which yielded the maximum protein amount. Solubility analysis showed that, under these conditions, the protein expressed in Escherichia coli BL21 was partitioned into both soluble (supernatant) and insoluble (pellet) fractions, with a target band at approximately 34 kDa (Figure 4C). The soluble fraction was then subjected to overexpression and purification. Western blot analysis using anti-His monoclonal antibody confirmed the successful expression of His-tagged Lysin K in *E. coli* BL21 (DE3) (Figure 4D). The final concentration of the purified endolysin was 100 μg/mL.

### 3.4. Enzymatic Characteristics and In Vitro Antibacterial Activity

**Figure 5:**
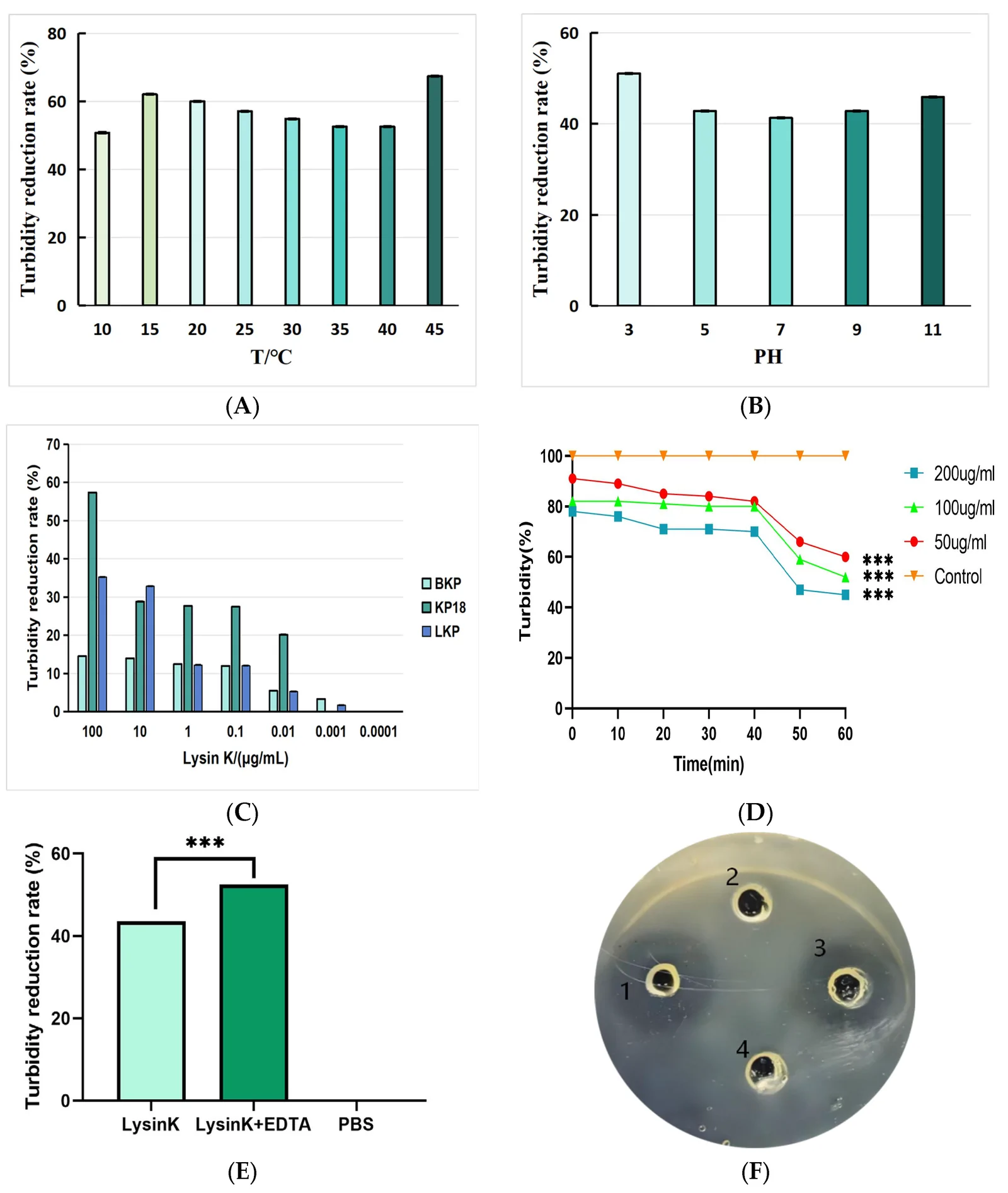

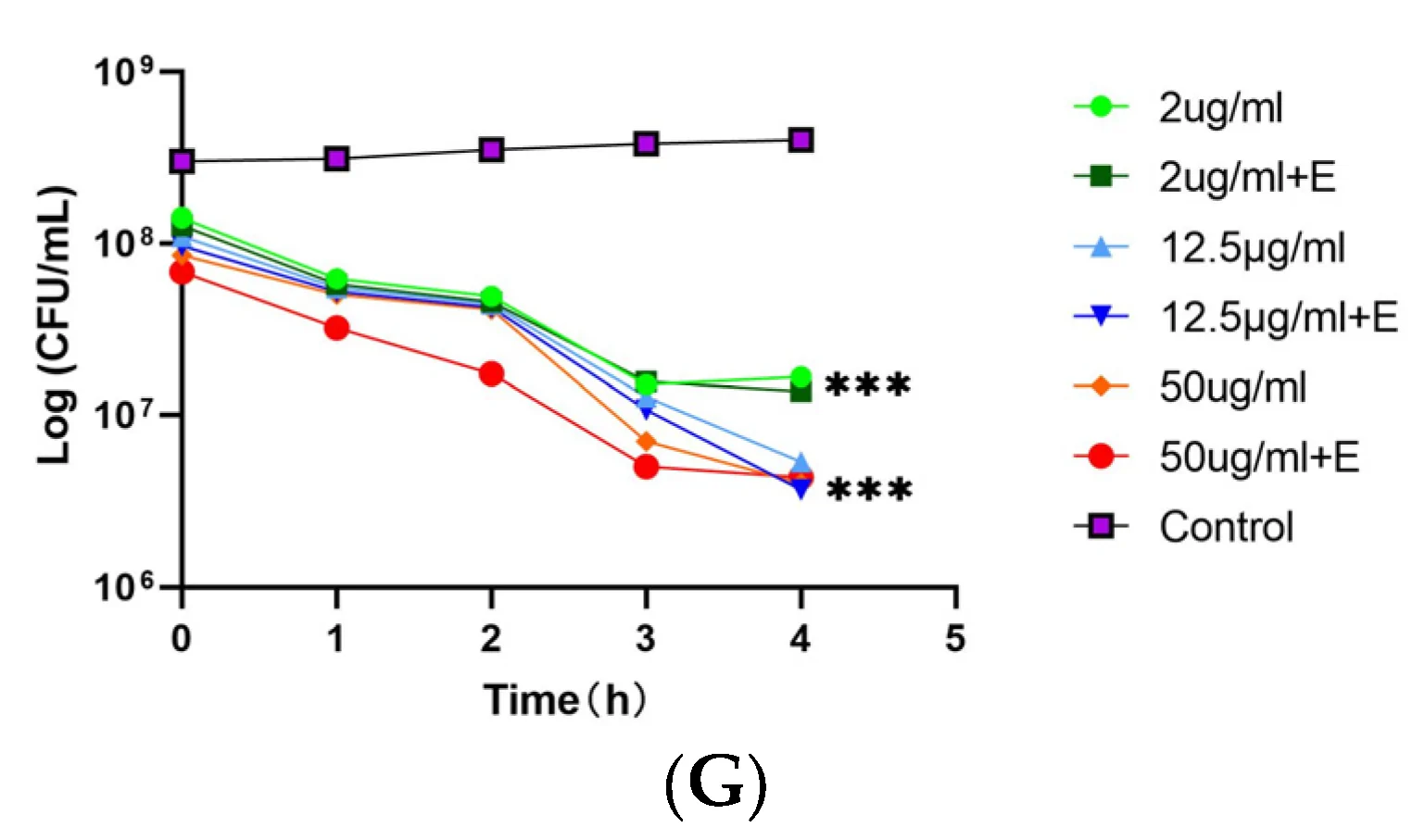
(**A**) Thermal stability analysis. (**B**) pH stability assays. (**C**) Minimum inhibitory concentration. (**D**) Time-kill curves. (**E**) Analysis of the modulatory effect of EDTA on Lysin K activity. (**F**) Antibacterial results of agar well diffusion assay 1, Lysin+EDTA; 2, EDTA; 3, Lysin; 4, Sterile PBS. (**G**) Antibacterial testing in milk. (*** *p* < 0.005; ns, not significant compared to the sterile PBS control group).

The host range assay revealed that lytic activity was only observed against strains LKP, BKP, and KP18; lysis was not observed for any other tested strain. Thermal stability analysis (Figure 5A) indicated that the optimal temperature for Lysin K activity was 45 °C, at which the turbidity reduction rate reached 67.40%. Moreover, Lysin K maintained strong antibacterial activity (50.77–62.05%) across the temperature range of 10–40 °C. pH stability assays (Figure 5B) revealed that Lysin K exerted the strongest lytic capacity at pH 3, with a turbidity reduction rate of 51.03%, and retained considerable inhibitory activity (41.28–45.90%) under pH conditions ranging from 5 to 11. Minimum inhibitory concentration (MIC) tests (Figure 5C) demonstrated that Lysin K possessed prominent lytic activity against all three tested *Klebsiella pneumoniae* strains. Stable antibacterial efficacy was observed even at an extremely low concentration of 0.01 μg/mL, and its lytic potency increased in a dose-dependent manner with rising protein concentration.

Time-kill curves (Figure 5D) confirmed that the lytic activity of Lysin K against *Klebsiella pneumoniae* KP18 exhibited time- and dose-dependent responses. Elevated Lysin K concentrations yielded enhanced bactericidal efficiency. After 1 h of incubation, the turbidity was reduced by 39.6% with 50 μg/mL Lysin K, while treatment with 200 μg/mL Lysin K produced a 55.1% turbidity reduction.

The analysis of the modulatory effect of EDTA on Lysin K activity (Figure 5E) showed that 0.5 mmol/L EDTA significantly boosted the lytic capacity of Lysin K, raising the turbidity reduction rate from 43.47% to 52.47%. Meanwhile, results from the agar well diffusion assay (Figure 5F) illustrated that Lysin K alone could suppress *Klebsiella pneumoniae* KP18 growth, generating an inhibition zone with a diameter of 0.80 cm. When combined with EDTA, the inhibition zone expanded to 1.00 cm, revealing a synergistic enhancement of bactericidal efficacy between the two agents.

Antibacterial testing in milk (Figure 5G) verified that Lysin K retained robust inhibitory activity in a milk matrix. After 4 h of incubation, treatments with 12.5 μg/mL and 50 μg/mL Lysin K both achieved approximately a 2-log_10_ reduction in viable bacterial counts in milk, whereas 2 μg/mL Lysin K yielded a roughly 1.3-log_10_ CFU reduction. The co-administration of Lysin K at all tested concentrations with EDTA accelerated the lysis rate.

### 3.5. Mouse Mastitis Model Establishment

#### 3.5.1. Clinical Observation

**Figure 6:**
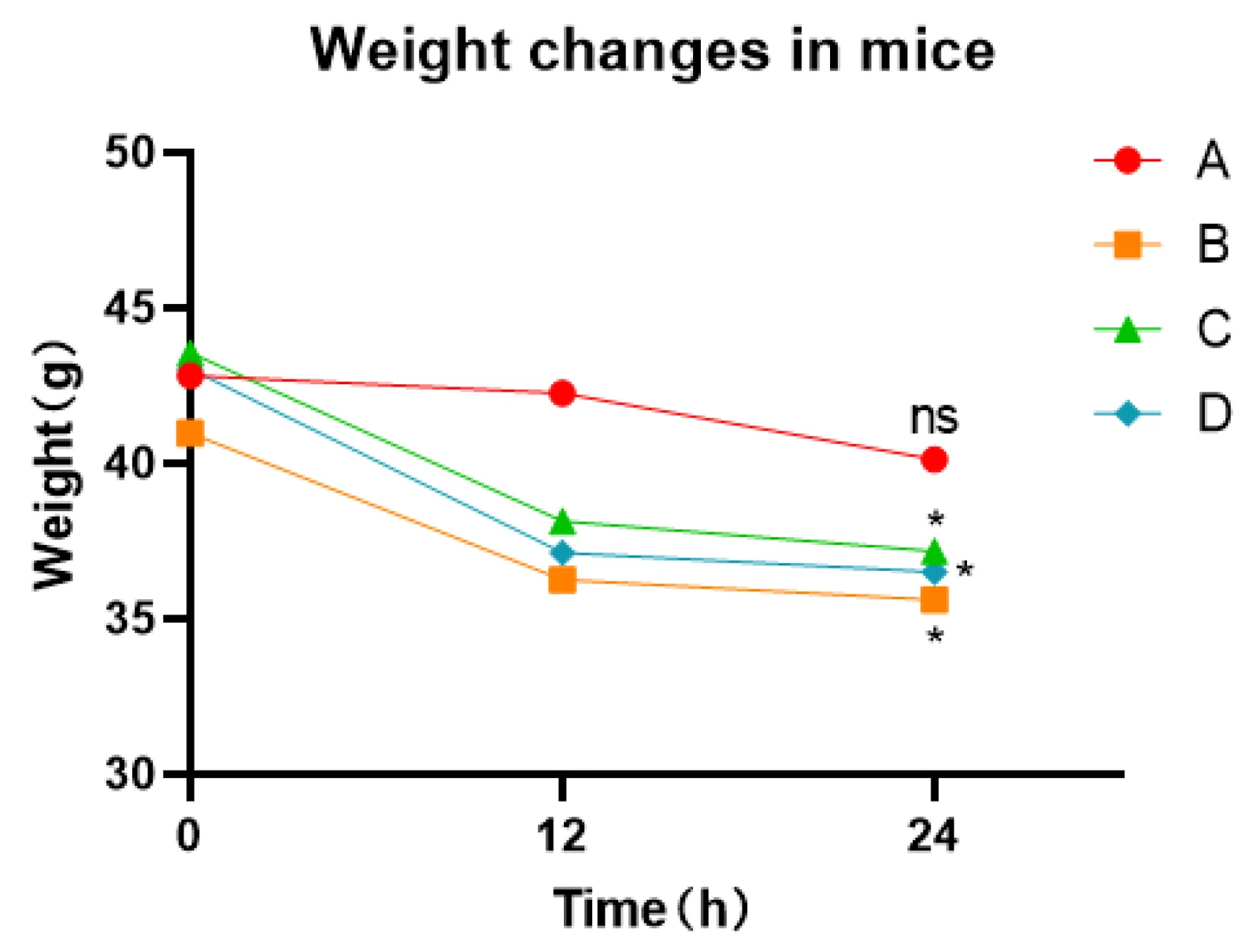
Body weight changes in mice. * *p* < 0.05; compared to the sterile PBS buffer control group at the same time point; ns, not significant. (A) Sterile PBS buffer control group. (B) 1.0 × 10^6^ CFU/mL bacterial suspension. (C) 1.0 × 10^8^ CFU/mL bacterial suspension. (D) 1.0 × 10^10^ CFU/mL bacterial suspension.

Following bacterial challenge, clinical signs and body weight were monitored at 12 h intervals. At 24 h post-challenge, all mice were euthanized, and mammary glands were collected for histopathological analysis. The results showed that the body weight of mice in all groups decreased. However, mice in the *Klebsiella pneumoniae*-challenged groups exhibited a significantly more pronounced weight loss compared with those in the sterile PBS control group (Figure 6).

Throughout the experimental period, mice in group A (sterile PBS control group) exhibited normal behavior, including clean fur and normal consumption of food and water. No mortality occurred. In contrast, mice in group B showed signs of depression and reduced feeding 12 h post-challenge, appearing lethargic and huddled, with warm, pink mammary glands. Mice in group C displayed depression, piloerection, and reduced consumption after 12 h, along with elevated body temperature and mammary gland swelling. By 24 h, they exhibited huddling behavior, reduced mobility, and erythematous, swollen mammary glands that were tender to palpation. Mice in group D exhibited severe depression, near-complete anorexia and adipsia, piloerection, shivering, and huddling at 12 h, along with elevated body temperature and red, swollen mammary glands. At 24 h, the glands showed significant swelling and marked tenderness upon palpation.

#### 3.5.2. Mammary Gland Necropsy

**Figure 7:**
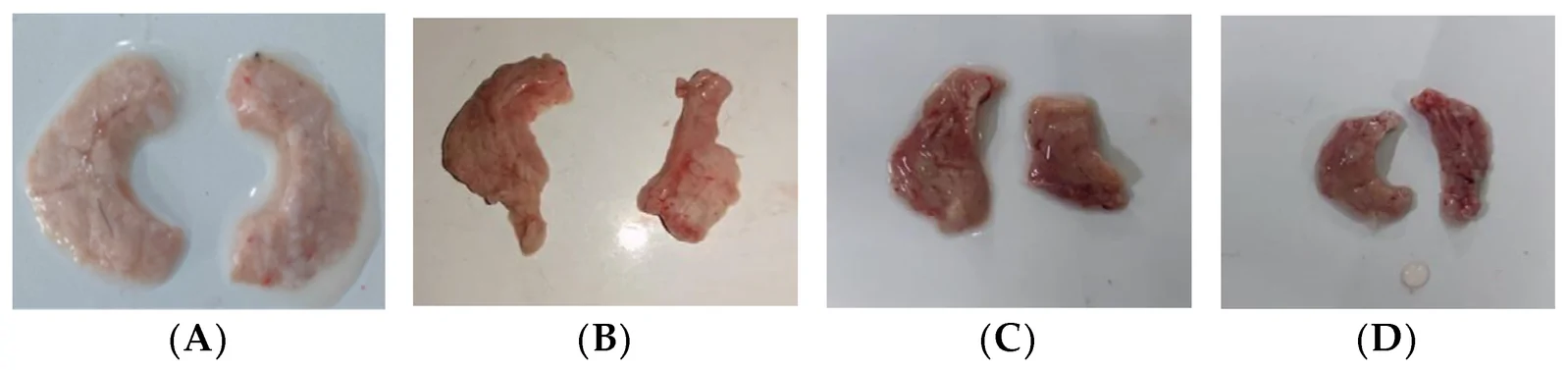
Gross examination of mammary glands from challenged groups. (**A**) Sterile PBS buffer control group. (**B**) 1.0 × 10^6^ CFU/mL bacterial suspension. (**C**) 1.0 × 10^8^ CFU/mL bacterial suspension. (**D**) 1.0 × 10^10^ CFU/mL bacterial suspension.

The results of the gross pathological examination of mammary glands are as follows. In group A, the glands were normal pinkish-white and soft, and no abnormalities were observed. In group B, the mammary glands appeared dark pink with peripheral hyperemia. In group C, they were erythematous, with a larger area of hyperemia compared to group B, and displayed multiple ecchymoses and petechial hemorrhages. In group D, the mammary glands were erythematous and firm, with multiple ecchymoses and hemorrhagic spots on the surface. These findings are consistent with reports from established mouse models [20,21], preliminarily confirming the successful establishment of the model (Figure 7).

#### 3.5.3. Analysis of Bacterial Load and IL-6 Levels

**Figure 8:**
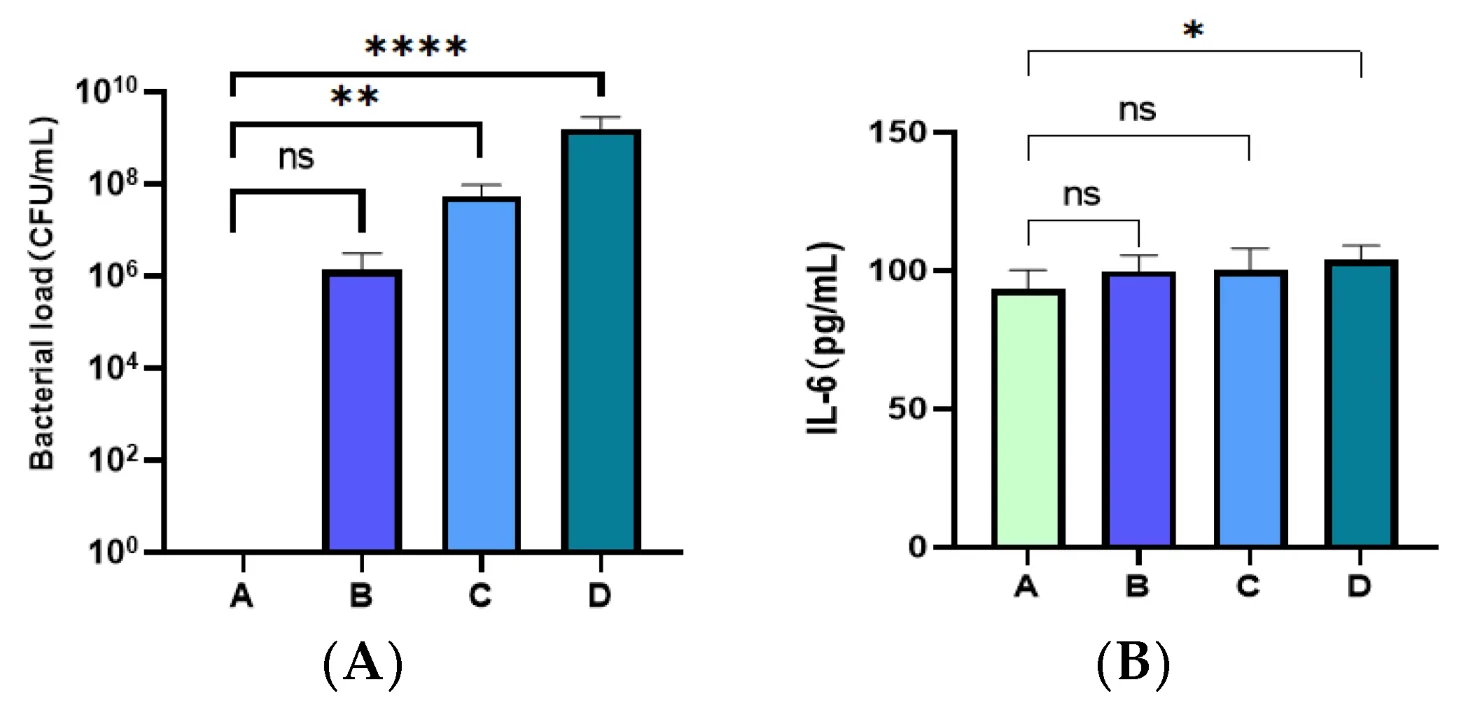
Bacterial counts in tissue homogenates and IL-6 concentrations. (**A**) Bacterial load in mammary tissues of challenged groups (**** *p* < 0.001; ** *p* < 0.01; ns, not significant compared to the sterile PBS control group data); (**B**) IL-6 concentration in mammary tissues of challenged groups (* *p* < 0.05; ns, not significant compared to the sterile PBS control group data).

The quantification of bacterial loads and IL-6 concentrations in tissue homogenates revealed that Group A exhibited neither detectable bacterial colonization nor elevated IL-6 levels. In contrast, the *Klebsiella pneumoniae* colony counts and IL-6 concentrations in infected groups increased correspondingly with the elevated bacterial challenge dose. Detailed results are shown in Figure 8.

#### 3.5.4. Pathological Analysis of Mammary Tissues

**Figure 9:**
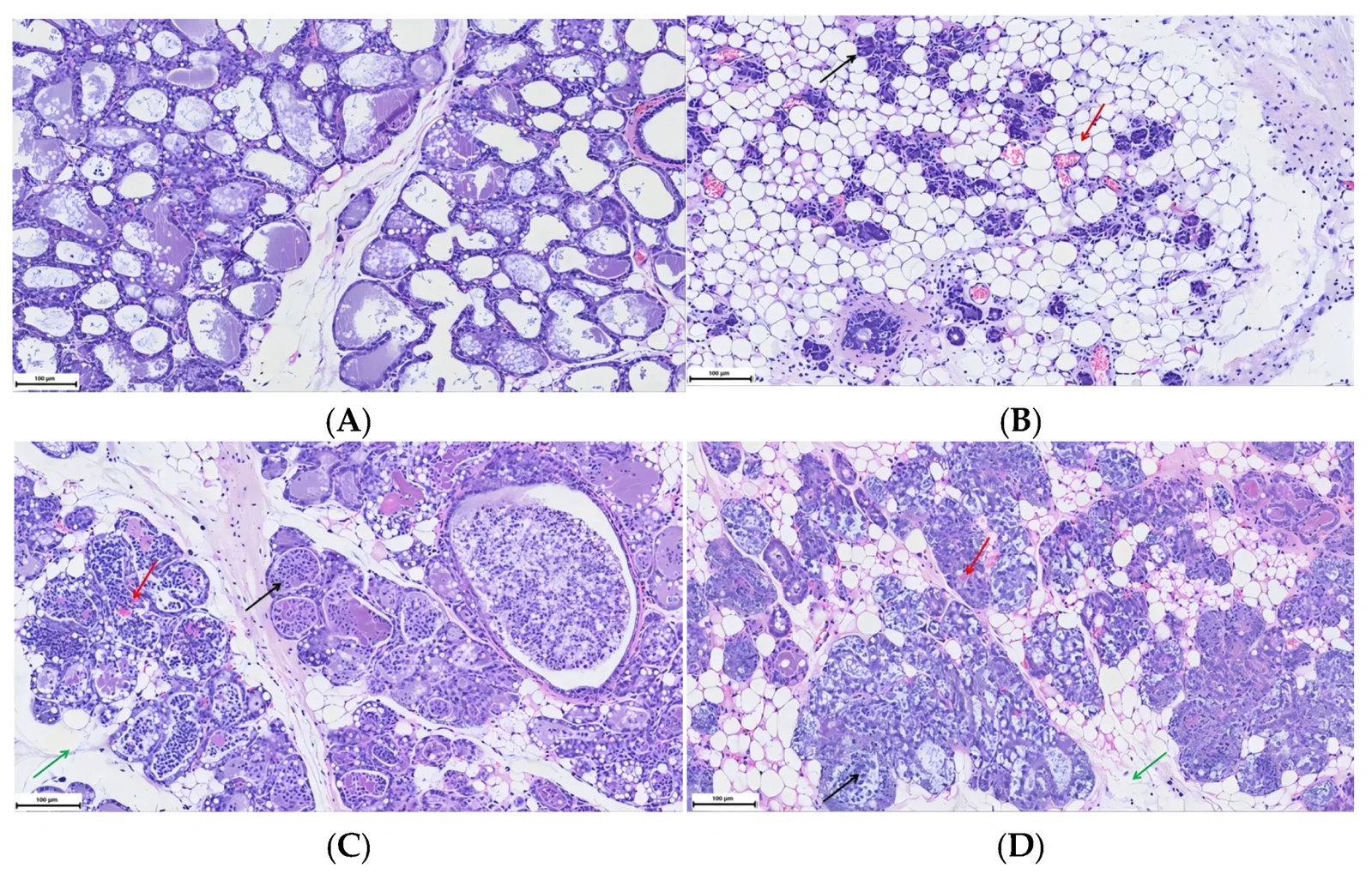
Pathological sections of mouse mammary tissues (H.E., staining): (**A**) sterile PBS buffer control group; (**B**) experimental group challenged with *Klebsiella pneumoniae* suspension at 1.0 × 10^6^ CFU/mL; (**C**) experimental group challenged with *Klebsiella pneumoniae* suspension at 1.0 × 10^8^ CFU/mL; (**D**) experimental group challenged with *Klebsiella pneumoniae* suspension at 1.0 × 10^10^ CFU/mL. Hemorrhage (red arrows); inflammatory cell infiltration (black arrows); disintegration of mammary architecture (green arrows).

The pathological analysis of mouse mammary tissue is shown in Figure 9. The mammary tissue in group A was structurally intact, with clearly defined alveolar walls and no obvious pathological alterations. In group B, a small number of red blood cells and neutrophil infiltration were observed within the mammary alveoli. Group C exhibited incomplete mammary tissue architecture with widespread disintegration, indistinct alveolar walls, and a massive infiltration of inflammatory cells (predominantly neutrophils). In group D, the alveolar structure was severely disrupted, mammary epithelial cells were largely absent, alveolar walls were indistinct, and the alveoli were filled with a massive influx of inflammatory cells.

In summary, compared with group A, mice challenged with *Klebsiella pneumoniae* exhibited dose-dependent pathological changes, including alveolar destruction and inflammatory cell infiltration. These histopathological effects increased bacterial loads, IL-6 concentrations, and breast injury score (Table 8), confirming the successful establishment of the mastitis model.

### 3.6. In Vivo Antibacterial Activity

**Figure 10:**
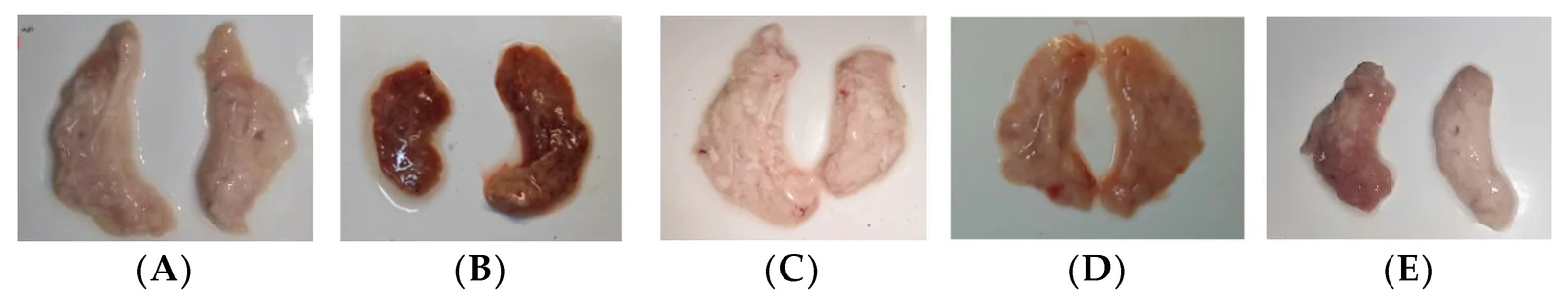
Gross examination results of mammary glands from mice in treatment groups. (**A**) Lysin+EDTA (0.5 mmol/L) control group. (**B**) Sterile PBS treatment group. (**C**) Antibiotic treatment group. (**D**) Lysin treatment group. (**E**) Lysin+EDTA (0.5 mmol/L) treatment group.

Gross pathological findings of the mammary glands are presented in Figure 10. Mammary glands in group A were normal pinkish-whiteand soft in texture, and exhibited no obvious abnormalities. Those in group B showed erythematous discoloration, severe congestion and hemorrhage, a friable texture, and diffuse collapse. In group C, the mammary glands were normal pinkish-white, with only a few hemorrhagic spots on the surface, and remained soft. In group D, the glands were dark pink and soft, with a small number of hemorrhagic spots and ecchymoses. In group E, the mammary glands were erythematous and soft, with mild congestion and a few hemorrhagic spots. Collectively, these gross pathological findings indicated that Lysin K, either alone or in combination with EDTA, alleviated the pathological manifestations of *Klebsiella pneumoniae*-induced mastitis in this model.

**Figure 11:**
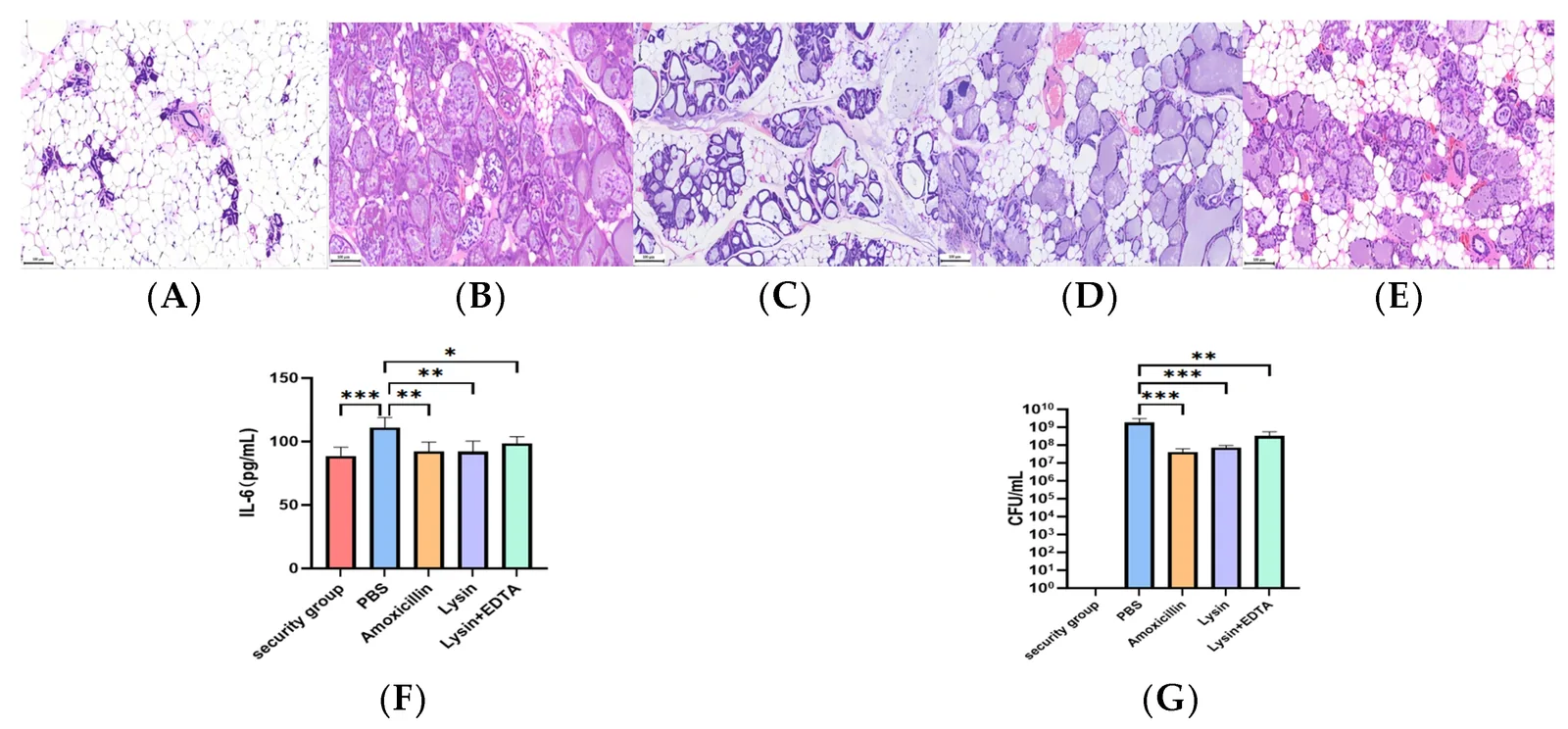
IL-6 concentration, bacterial load, and histopathological sections (H.E., staining) in mammary tissues of mice from treatment groups. (**A**) Lysin+EDTA control group. (**B**) Sterile PBS treatment group; (**C**) Antibiotic treatment group; (**D**) Lysin treatment group. (**E**) Lysin+EDTA treatment group. (**F**) IL-6 concentration in mammary tissues of treatment groups (* *p* < 0.05; ** *p* < 0.01; *** *p* < 0.005 compared to the sterile PBS treatment control group). (**G**) Bacterial load in mammary tissues of treatment groups (** *p* < 0.01; *** *p* < 0.005 compared to the sterile PBS treatment control group).

Pathological sections of mouse mammary tissue are shown in Figure 11A–E. The therapeutic efficacy of the phage endolysin Lysin K against mastitis was evaluated in a mouse model by assessing the severity of mammary lesions, as well as IL-6 cytokine levels (Figure 11A), bacterial burden (Figure 11B), and histopathological changes. Group A presented normal mammary tissue with intact alveoli, no disintegration, and no inflammatory cell infiltration. IL-6 concentration was the lowest among all groups, and no bacteria were detectable, confirming the safety of the formulations. Group B showed extensive inflammatory cell infiltration (predominantly neutrophils), a marked disruption of alveolar walls, and severe congestion. Group C exhibited largely intact mammary alveoli with only mild disintegration and a few red blood cells. Both IL-6 concentration and bacterial load were significantly decreased in this group. In group D, the mammary tissue was markedly more intact than in group B, with no obvious disintegration; only mild hyperemia, partial alveolar dilation, and a small amount of secretion were observed. IL-6 concentration was not significantly different from that in group C, while the bacterial load was significantly lower than in group B. Group E showed pathological changes similar to group D. Although the bacterial load and IL-6 concentration in this group were slightly higher than those in group D, they were still significantly lower than those in group B. The combination treatment also effectively reduced the bacterial burden and alleviated the inflammatory response. In conclusion, combined with mammary lesion scores (Table 9), Lysin K significantly inhibited the growth of Klebsiella pneumoniae and alleviated inflammatory responses in mouse mammary glands, representing a promising novel biological agent for the treatment of mastitis caused by this pathogen.

## 4. Discussion

At present, the overuse of antibiotics remains prevalent in animal husbandry. Multidrug-resistant *Klebsiella pneumoniae* (MDR-KP) and hypervirulent *Klebsiella pneumoniae* have become major risk factors contributing to bovine mastitis. Owing to their high host specificity, evolutionary adaptability, capacity for in situ amplification, and evolutionary trade-off mechanisms, bacteriophages exhibit prominent advantages in the prevention and control of multidrug-resistant bacteria, serving as promising alternative therapies for MDR-KP infections [22]. Multiple studies [23,24,25] have verified that recombinantly expressed phage endolysins can efficiently lyse both Gram-negative and Gram-positive bacteria. In the present study, the endolysin gene sequence of a *Klebsiella pneumoniae* phage isolated from a dairy farm in Sichuan Province was analyzed, and its conserved domains were predicted. Using the complete phage genome as the template, the endolysin Lysin K was heterologously expressed in a prokaryotic system. Subsequently, comprehensive analyses were conducted to characterize its lytic spectrum, thermal stability, pH stability, minimum inhibitory concentration (MIC), synergistic effects with EDTA, and antibacterial performance in milk. The protein retained antibacterial activity across 10–45 °C, a temperature range covering the typical body temperatures associated with animal *Klebsiella pneumoniae* infections. pH stability assays demonstrated that Lysin K maintained lytic activity at pH values ranging from 3 to 11, indicating its suitability for freshwater, seawater, and the weakly alkaline microenvironment of the bovine mammary gland; it even holds the potential to withstand the gastric acid barrier for oral administration. Furthermore, Lysin K exerted stable lytic effects against three *Klebsiella pneumoniae* strains at an extremely low MIC of 0.01 μg/mL and remained functionally stable in milk, highlighting its great potential for the prevention and treatment of bovine mastitis caused by *Klebsiella pneumoniae*. Notably, turbidity assays and agar well diffusion tests demonstrated that supplementation with the outer membrane permeabilizer EDTA markedly potentiated the antibacterial efficacy of Lysin K compared with Lysin K treatment alone. This observation is consistent with the intrinsic property that endolysins primarily target the peptidoglycan layer of bacterial cell walls [26,27]. Nevertheless, the outer membrane, composed of lipopolysaccharides (LPS), lipoproteins, porins and phospholipids, resides outside the cell wall of *Klebsiella pneumoniae* [28], which impedes endolysin penetration through the hydrophobic outer membrane to reach the peptidoglycan substrate. Previous research has proven that recombinant endolysins fused with membrane-penetrating peptides can traverse the outer membrane barrier and achieve satisfactory bactericidal activity. Lu et al. [29] constructed LysCA, a *Klebsiella pneumoniae* phage endolysin fused with cecropin A residues. Both in vitro and in vivo antibacterial assays showed that LysCA possessed substantially stronger lytic capacity and environmental tolerance than the unmodified endolysin LysG24, and effectively alleviated tissue lesions in a murine pneumonia model. In addition, numerous studies [30,31,32] have confirmed that outer membrane permeabilizers such as EDTA can enhance antibacterial activity. EDTA chelates divalent cations Mg^2+^ and Ca^2+^, which destabilizes the outer membrane, loosens lipopolysaccharide (LPS) architecture, and creates channels for endolysins to access their substrate. Ni et al. [33] reported that EDTA at concentrations of 0.5–10 mmol/L significantly improved the lytic activity of the *Vibrio alginolyticus* phage lysin LysV208 against its host strain, with activity elevated from 58.6% to 68.0%. The maximum lytic activity (68.0%) was achieved when combined with 0.5 mmol/L EDTA, representing a 2.8-fold increase relative to the group treated with lysin alone (24.2%). In this study, 0.5 mmol/L EDTA also enhanced the lytic activity of Lysin K by 17.15%, which was consistent with the findings reported in the above-mentioned studies.

In this study, the results showed that tissue injury in all treatment groups was significantly alleviated compared with the PBS group. Moreover, the clinical manifestations, tissue injury, bacterial load, and IL-6 concentration in the Lysin K-treated group were comparable to those in the amoxicillin-treated group, both of which exerted favorable therapeutic effects. The Lysin K+EDTA group confirmed that the combination exhibited no toxicity toward mouse mammary gland tissue. However, IL-6 concentration in the Lysin K+EDTA group was higher than that in the Lysin K monotherapy group. We speculated that this phenomenon would occur because, under pathological conditions, EDTA chelates the calcium ions required for intercellular junctions, leading to a mild disruption of tight junctions in mammary epithelial cells and the activation of local immune cells to secrete pro-inflammatory cytokines such as IL-6, thereby increasing local IL-6 levels [34]. Nevertheless, this effect did not induce severe tissue damage. Instead, it may promote the recruitment of local immune cells, accelerate the clearance of residual bacteria and cellular debris, and thus contribute to improved therapeutic outcomes. Furthermore, compared with the sterile PBS-treated control group, the Lysin K+EDTA–treated group exhibited decreased bacterial load, IL-6 concentration, and tissue damage. This result is consistent with the findings from existing clinical therapeutic studies [35,36,37].

Based on the combined in vitro and in vivo results, Lysin K shows therapeutic potential against mastitis caused by *Klebsiella pneumoniae*. Some phages carry genes encoding bacterial toxins and resistance determinants, which can lead to enhanced bacterial virulence and the horizontal transfer of drug resistance [38,39,40]. Therefore, the application of native phages for the treatment of *Klebsiella pneumoniae*-induced mastitis still carries potential risks. Numerous studies [41,42,43] have demonstrated that endolysins can lyse bacteria efficiently, are less prone to induce resistance, and exert no toxic effects on animal cells, making them more suitable for widespread application. Although phage endolysins may not completely eliminate pathogens, they can significantly reduce pathogen burden and assist the host immune system in clearing infections [44,45]. While antibiotic therapy may ultimately be necessary for the treatment of *Klebsiella pneumoniae* mastitis, the improvement of early clinical signs can allow dairy cows to recover productivity more rapidly, reduce the abuse of antibiotics, and help to address the global antimicrobial resistance crisis. In addition to the above strategies, existing studies have demonstrated that engineering artificial lysins or performing amino acid substitutions can enhance the lytic activity of lysins. Briers et al. [46] fused holin and a polypeptide fragment with a *Helicobacter pylori* phage lysin to construct a novel engineered lysin. Yan et al. [47] introduced hydrophobic amino acid residues into an *Escherichia coli* phage endolysin; the modified endolysin exhibited markedly improved bactericidal activity and an expanded lytic spectrum relative to the wild-type phage and its native endolysin. Furthermore, phage–antibiotic combination therapy represents another viable therapeutic strategy [48,49]. Phage endolysins can target bacterial biofilms and outer membranes, thereby facilitating the action of antibiotics; the evolutionary trade-off between bacterial resistance to phages and antibiotics reduces the likelihood of bacteria developing tolerance to both agents simultaneously. Therefore, investigating the interactions between bacteria and phage lytic proteins, as well as optimizing combination regimens of phage endolysins and antibiotics, is expected to provide solid theoretical and experimental support for the prevention and control of drug-resistant *Klebsiella pneumoniae* and the treatment of bovine mastitis caused by this pathogen.

## 5. Conclusions

In summary, this study demonstrates that recombinantly expressed and purified endolysin Lysin K exhibits potent antibacterial activity against multidrug-resistant *Klebsiella pneumoniae* in both in vitro and in vivo settings. Endolysin Lysin K was biocompatible in the murine mastitis model, supporting its potential as a novel therapeutic candidate for mastitis caused by drug-resistant *Klebsiella pneumoniae*.

## Figures and Tables

**Table 1:** Primer information.

Prime	Genes	Sides
Holin	F:CCGGAATTCATGGATAAATTTATACAG	663 bp
	R: CAAGCTTTCATATTGATCTCCC	
Lysin K	F:GGAATTCATGAGTTTCAGATTTGGAAAC	414 bp
	R: CAAGCTTTCACAGTAACTCTACGTGTC	
M13	F: CGCCAGGGTTTTCCCAGTCACGAC R:AGCGGATAAcAATTTCACACAGGA	n + 200 bp
T7	F: CCCGCGAAATTAATACGACT R: GCTAGTTATTGCTCAGCGG	693 bp

**Table 2:** Optimization of Lysin K induction conditions.

Induced Temperature (°C)	Induction Time (h)	IPTG Concentration (mmol/L)
16	0	0.4
	2	0.6
25	4	0.8
30	6	1.0
37	8	1.2

**Table 3:** Strains used for cleavage spectrum test.

Number	Bacteria	Host
1	*E. coli O157:H7*	Cattle
2	*Escherichia coli* *K12*	Goats
3	*Citrobacter freundii*	Cattle
4	*Klebsiella pneumoniae*	Cattle
5	*Klebsiella pneumoniae*	Musk deer
6	*Klebsiella mutans*	Cattle
7	*Salmonella gallinis*	Chickens
8	*Salmonella muris*	Mice
9	*Salmonella bovis*	Cattle
10	*Shigella SE6-1*	Cattle
11	*Shigella SWHEFF 49*	Cattle
12	*Staphylococcus sciuri*	Goats
13	*Staphylococcus aureus*	Cattle

**Table 4:** Grouping of in vitro antibacterial activity detection.

Grouping Situation	Sample Addition Situation
1	30 μL Lysin K+EDTA (0.5 mmol/L)
2	30 μL EDTA (0.5 mmol/L)
3	30 μL Lysin K (100 μg/mL)
4	30 μL PBS

**Table 5:** Grouping of experimental animals.

Group	Reagent	Dose (μL)
A	sterile PBS	100 μL/nipple
B	1.0 × 10^6^ CFU/mL bacterial suspension	100 μL/nipple
C	1.0 × 10^8^ CFU/mL bacterial suspension	100 μL/nipple
D	1.0 × 10^10^ CFU/mL bacterial suspension	100 μL/nipple

**Table 6:** Grouping of mice.

Group	Type	Reagent Name
A	Lysin K+EDTA (0.5 mmol/L) group	PBS-containing Lysin K+EDTA
B	control group	Sterile PBS
C	Antibiotic group	Amoxicillin (50 μg/mL)
D	Lysin K group	PBS-containing Lysin K (protein concentration: 100 μg/mL)
E	Lysin K+EDTA (0.5 mmol/L) group	PBS-containing Lysin K+EDTA (protein concentration: 100 μg/mL)

**Table 7:** Conserved domains of the lysin.

Name	Accession	Description	Interval	E-Value
L−Ala−D−Glu_peptidase_like	cd14845	L−Ala−D−Glu_peptidase, also known as L−alanyl−D−glutamate endopeptidase	7−390	2.80 × 10^−20^
Peptidase_M15_4	Pfam13539	D−alany-D−alanine carboxypeptidase	55−225	2.27 × 10^−4^
Peptidase_M15_like	cd14846	Uncharacterized family of the peptidase family M15, subfamily B	103−366	1.58 × 10^−3^
Peptidase_M15	cd14814	Metalloproteases, including zinc D-Ala-D-Ala carboxypeptidase, L−Ala−D−Glu peptidase, L, D−carboxypeptidase, bacteriophage endolysins, and related proteins	7−363	6.60 × 10^−3^

**Table 8:** Grading of histopathological features in the mammary of mice (KP18-challenged group).

Group	Congestion and Edema	Mammary Acinar Dilatation	Neutrophilic Infiltration
A	0	0	0
B	1	1	1
C	1	2	2
D	2	3	3

No lesions or very few lesions were scored as 0; slight lesions were scored as 1; moderate lesions were scored as 2; severe lesions were scored as 3.

**Table 9:** Grading of histopathological features in the mammary of mice (Lysin K treatment group).

Group	Congestion and Edema	Mammary Acinar Dilatation	Neutrophilic Infiltration
A	0	0	0
B	3	3	3
C	0	0	0
D	1	1	0
E	1	2	1

No lesions or very few lesions were scored as 0; slight lesions were scored as 1; moderate lesions were scored as 2; severe lesions were scored as 3.

## Data Availability

The repository name and accession numbers in the study are openly available in GenBank at https://www.ncbi.nlm.nih.gov/genbank/, reference number MT380195.1. (accessed on 25 April 2025)
